# Improving accuracy for finite element modeling of endovascular coiling of intracranial aneurysm

**DOI:** 10.1371/journal.pone.0226421

**Published:** 2019-12-27

**Authors:** Robert J. Damiano, Vincent M. Tutino, Saeb R. Lamooki, Nikhil Paliwal, Gary F. Dargush, Jason M. Davies, Adnan H. Siddiqui, Hui Meng

**Affiliations:** 1 Department of Mechanical and Aerospace Engineering, University at Buffalo, State University of New York, Buffalo, New York, United States of America; 2 Canon Stroke & Vascular Research Center, University at Buffalo, State University of New York, Buffalo, New York, United States of America; 3 Department of Neurosurgery, University at Buffalo, State University of New York, Buffalo, New York, United States of America; 4 Department of Pathology and Anatomical Sciences, University at Buffalo, State University of New York, Buffalo, New York, United States of America; 5 Department of Biomedical Engineering, University at Buffalo, State University of New York, Buffalo, New York, United States of America; University of Michigan, UNITED STATES

## Abstract

**Background:**

Computer modeling of endovascular coiling intervention for intracranial aneurysm could enable *a priori* patient-specific treatment evaluation. To that end, we previously developed a finite element method (FEM) coiling technique, which incorporated simplified assumptions. To improve accuracy in capturing real-life coiling, we aimed to enhance the modeling strategies and experimentally test whether improvements lead to more accurate coiling simulations.

**Methods:**

We previously modeled coils using a pre-shape based on mathematical curves and mechanical properties based on those of platinum wires. In the improved version, to better represent the physical properties of coils, we model coil pre-shapes based on how they are manufactured, and their mechanical properties based on their spring-like geometric structures. To enhance the deployment mechanics, we include coil advancement to the aneurysm in FEM simulations. To test if these new strategies produce more accurate coil deployments, we fabricated silicone phantoms of 2 patient-specific aneurysms in duplicate, deployed coils in each, and quantified coil distributions from intra-aneurysmal cross-sections using coil density (*CD*) and lacunarity (*L*). These deployments were simulated 9 times each using the original and improved techniques, and *CD* and *L* were calculated for cross-sections matching those in the experiments. To compare the 2 simulation techniques, Euclidean distances (*d*_*Min*_, *d*_*Max*_, and *d*_*Avg*_) between experimental and simulation points in standardized *CD*-*L* space were evaluated. Univariate tests were performed to determine if these distances were significantly different between the 2 simulations.

**Results:**

Coil deployments using the improved technique agreed better with experiments than the original technique. All *d*_*Min*_, *d*_*Max*_, and *d*_*Avg*_ values were smaller for the improved technique, and the average values across all simulations for the improved technique were significantly smaller than those from the original technique (*d*_*Min*_: *p* = 0.014, *d*_*Max*_: *p* = 0.013, *d*_*Avg*_: *p* = 0.045).

**Conclusion:**

Incorporating coil-specific physical properties and mechanics improves accuracy of FEM simulations of endovascular intracranial aneurysm coiling.

## Introduction

Coil embolization is the primary endovascular therapy for intracranial aneurysms (IAs). Coils are metallic devices that are implanted into the IA sac in order to induce thrombotic occlusion and exclude the aneurysm from the cerebral circulation. However, many coiled IAs that are initially occluded after treatment are found to be recanalized (unoccluded) on follow-up imaging (20.8%, mean 14.1 months), and many that are *not* initially occluded remain so at follow-up (16.6%, mean 14.1 months) [1]. Coiled IAs that reacanalize or do not fully occlude remain susceptible to the risk of rupture or complications from difficult re-treatments [2]. If clinicians could predict the outcome of a particular coil treatment *a priori*, unfavorable outcomes could be avoided.

Since coils modify intra-aneurysmal flow, treatment outcomes likely depend on both the deployed coil configuration and the post-treatment aneurysmal hemodynamics. However, in routine clinical practice, this information is not available. Detailed coil configurations cannot be resolved on imaging, such as digital subtraction angiography (DSA) or computer tomography angiography (CTA). Because the coils obscure the IA in images, post-treatment aneurysmal hemodynamics also cannot be obtained directly from imaging. To this end, virtual intervention, i.e. computationally modeling endovascular coiling and hemodynamics for specific patients, has emerged as a promising option for detailed evaluation of the deployed coils and aneurysmal flow.

Currently, the best approach to accurately model deployed coil configurations (and therefore post-treatment hemodynamics) may be the finite element method (FEM). This is because FEM explicitly models the physical properties of coils, i.e. geometry and mechanical properties, and the mechanics of the deployment procedure [3–7]. For computational efficiency, previous FEM techniques simplified the mechanics of coil deployment. One notable simplification was the bypass of packaging the coil into the catheter [4, 7]. Coil packaging creates stored strain energy that drives the coil to recover its pre-shape during deployment; therefore skipping this step could result in unphysical deployments.

Recently, we developed an FEM-based coiling technique that included packaging the coil into the catheter [3], and applied it along with computational fluid dynamics (CFD) to investigate the hemodynamic effects of several coil treatment strategies. However, this technique still included several simplifications: (1) the coil pre-shape was modeled using a generalized mathematical curve [4]; (2) the coil mechanical properties were modeled using those of a platinum wire [4]; and (3) the advancement of the packaged coil in the catheter through the artery was omitted (coil was simply released perpendicularly at the IA neck). We suspect these simplifying assumptions will ultimately hamper the goal of accurate patient-specific virtual intervention, which requires realistic simulations to recapitulate patient-specific coiling cases.

The objective of the current study is to improve the modeling assumptions in our FEM coiling technique. For a realistic coil pre-shape, we account for the coil manufacturing process by “virtually manufacturing” the coil–modeling a mandrel and winding the coil around it to obtain a pre-shape. For realistic mechanical properties of coils with different stiffness, we adopt mechanical rigidity of spring-like structures, following an approach by Otani et al. [5]. Both of these improved assumptions allow the physical properties of a wide array of coils to be modeled. Moreover, we explicitly simulate advancing the coil through the parent artery.

Here, we describe these key strategies for improving the FEM coiling technique and present the overall virtual coiling workflow. Furthermore, to test whether implementing these strategies result in improved accuracy of coil deployments, we compare simulations of coil deployment using our previously-developed technique and our new technique against *in vitro* experimental results.

## Methods

The institutional review board at University at Buffalo approved this study (study no. 030–510704). Patient consent was waived and all data and images were collected retrospectively and de-identified. All methods were conducted in accordance with the approved protocol.

### Key strategies to improve the FEM coiling technique

Below, we describe the key improvements we made to the FEM coiling technique (our “Improved Technique”) as compared to the “Original Technique”, and then present the entire workflow for simulating coil deployment in patient-specific IAs. We improved the modeling strategies for 3 specific components of the FEM technique: coil geometry, coil mechanical properties, and the mechanical steps of coiling.

#### Modeling strategies for coil geometry

**Fig 1:**
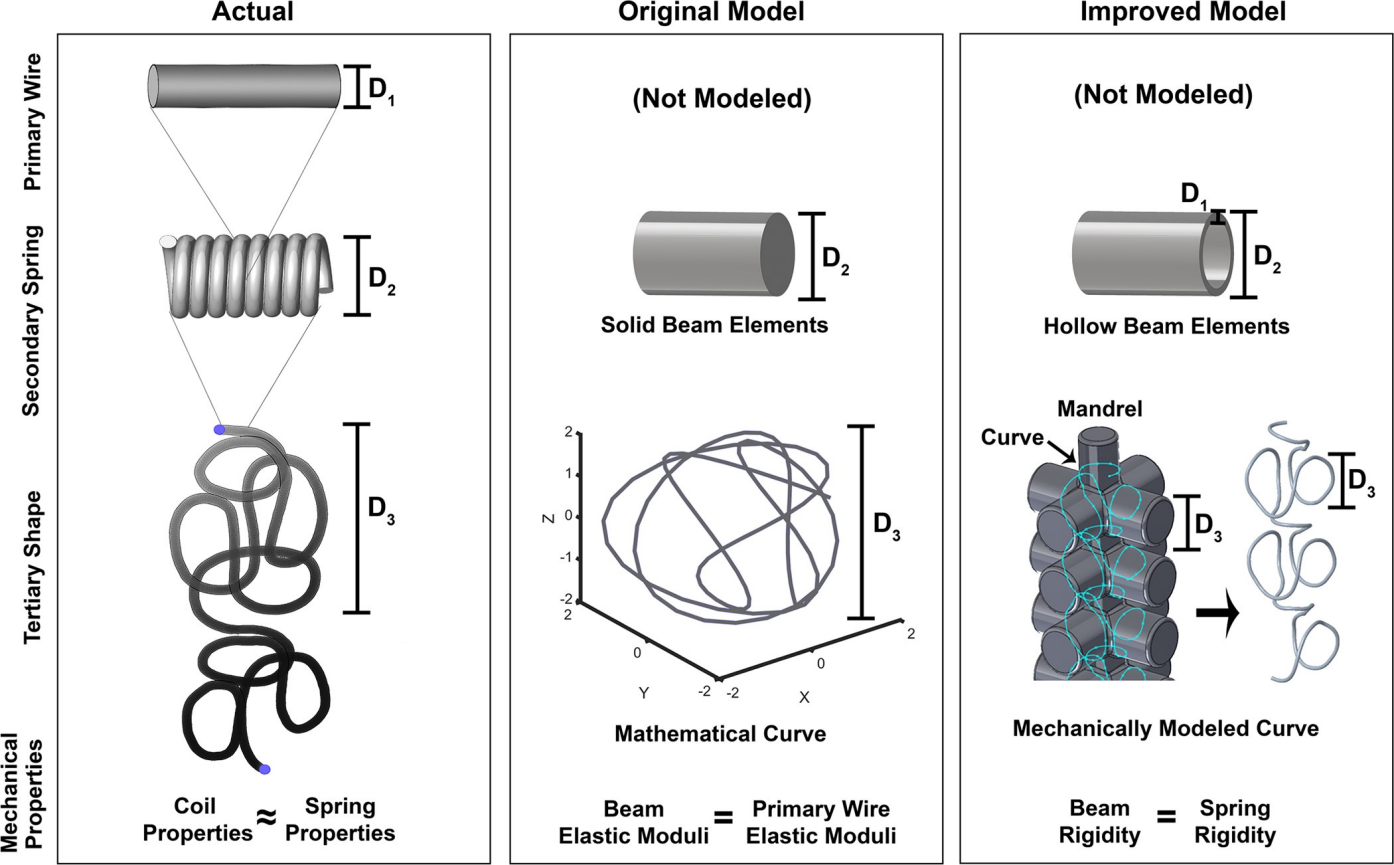
The physical properties of coils and how they were modeled. Top Row: The coil’s primary stock wire is composed of platinum and was not modeled in either technique. 2nd Row: The coil’s secondary structure comprises the stock wire wound to form a helical spring-like structure. The secondary structure was modeled as a series of beam elements in both techniques, with the Improved Technique using hollow elements and the Original using solid elements. 3rd Row: The coils tertiary “pre-shape” is created when the spring-like structure is wound around a mandrel and heat-treated to create a 3D shape with “shape-memory”. The Original Technique modeled the pre-shape as mathematical curves generated from parametric equations, while the Improved Technique modeled the pre-shape by virtually winding the coil’s secondary structure around a mandrel. Bottom Row: We modeled coil mechanical properties after springs, with the Original Technique assuming beam elements had the elastic moduli of a platinum wire and the Improved Technique calculating beam properties by equating beam rigidity to spring rigidity.

Coils have a multiscale structure, a primary cylindrical wire (diameter *D*_1_, elastic moduli *E*_*w*_ and *G*_*w*;_ *w* = *wire*), a secondary spring-like structure (diameter *D*_2_, number of primary wire loops *n*, length *l*), and a 3D tertiary structure (diameter *D*_3_), as illustrated in the left column of Fig 1. The 3D tertiary structure results from winding the coil around a complex-shaped mandrel and heat-treating it [8]. Pulling the coil from its tertiary structure, or “pre-shape”, into the catheter during packaging causes it to store strain energy, which later drives the coil to recover this pre-shape during deployment.

In the Original Technique [3], the primary wire is not modeled and the secondary structure is modeled as a series of solid Euler-Bernoulli beam elements with diameter *D*_2_ (middle column of Fig 1). In the Improved Technique, the primary wire is also not modeled, but to account for its geometry (i.e. *D*_1_), the secondary structure is modeled as a series of tubular, Timoshenko beam elements with thickness *D*_1_ and outer diameter *D*_2_ (right column of Fig 1). We use Timoshenko beam elements to model coils in the Improved Technique, instead of Euler-Bernoulli beams (used in the Original Technique), because they are more appropriate if fine mesh discretizations consisting of short beams are required to resolve contact, especially in complex cases such as deployment of several long coils into a large IA. In addition, Timoshenko beam elements are more appropriate than Euler-Bernoulli beam elements to handle large coil deformations that could occur in such complex treatment cases.

To model the tertiary pre-shape, the Original Technique uses generalized mathematical curves with pre-shape diameter *D*_3_ (middle column of Fig 1), generated from 3D parametric equations derived by Babiker et al. [4]. To model coil pre-shape more realistically in the Improved Technique, we “virtually manufacture” it by modeling a mandrel, and then winding the coil around the mandrel, resulting in the pre-shape. The mandrel geometry is generalized from actual mandrels [8], and is modeled in Solidworks (ver. 2016, Dassault Systèmes, Vélizy-Villacoublay, France) as a main cylinder of diameter *D*_3_−1.5 *mm* with several cross-axis cylinders of diameter *D*_3_ connected to it (right column of Fig 1). We mimic winding the coil around the mandrel by winding a B-spline curve along the surface of the mandrel using the “spline on surface” feature in Solidworks. The B-spline curve is wound around the main and cross-axis cylinders of the mandrel such that the curve does not intersect with itself. The B-spline is truncated to have the same length as the coil and it becomes the centerline of the coil’s beam elements in the FEM simulations.

#### Modeling strategies for coil mechanical properties

For coil mechanical properties, the Original Technique assumes that the elastic moduli of the coil’s beam elements (i.e. *E*_*b*_, *G*_*b*_;*b* = *beam*) are the same as the coil’s primary wire (*E*_*w*_ and *G*_*w*_), i.e. platinum. In the Improved Technique, we adopt mechanical properties of helical springs to model the properties of coils because coils and springs are geometrically similar [9–11]. To do so, we follow the approach of Otani et al. by determining the appropriate elastic moduli of the coil’s beam elements, such that the rigidity of the beam is equivalent to that of the helical spring-like coil [5, 11]. While this approach already appears in the literature, it represents an important modification from our Original Technique [3], in terms of model fidelity and computational efficiency. The mechanical rigidities of a helical spring–namely compressive (*D*_*compressive*_), shearing (*D*_*shearing*_), and flexural (*D*_*flexural*_)–are determined by its geometry and the material properties of its primary wire [11]:

$$D_{compressive}=\frac{G_{w}D_{1}^{4}l}{8nD_{2}^{3}}$$ (1)

$$D_{shearing}=\frac{E_{w}D_{1}^{4}l}{8nD_{2}^{3}}$$ (2)

$$D_{flexural}=\frac{E_{w}{G_{w}D}_{1}^{4}l}{16nD_{2}(2G_{w}+E_{w})}$$ (3)

The appropriate elastic moduli for the coil’s beam elements are calculated by equating beam rigidity to spring rigidity:

$$E_{b}A_{b}=D_{compressive}$$ (4)

$${\lambda G}_{b}A_{b}=D_{shearing}$$ (5)

$$E_{b}I_{b}=D_{flexural}$$ (6)

Here, *A*_*b*_ is the beam’s cross-section area, *I*_*b*_ is its area moment, and *λ* is a shape correction factor, which is approximately 0.5 for a tubular beam [12]. Assuming that the coil’s primary wire loops are tightly wound, we approximate the length of the coil (*l*) to be *nD*_1_.

#### Modeling strategies for the mechanical steps of coiling

In terms of the mechanics of coil deployment, 3 steps play key roles in determining the final configuration of a deployed coil: *coil packaging* into the catheter, *coil advancement* through the parent artery in the coil-loaded catheter, and *coil deployment* into the IA. Done by the manufacturer, *coil packaging* is a critical mechanical step that determines the coil’s behavior when released from the catheter during *coil deployment* because packaging causes the coil to store strain energy that drives it to recover its pre-shape.

In our Original Technique, coil packaging and coil deployment are modeled, but not coil advancement. To bypass coil advancement, we isolate the IA sac from its parent artery, and directly place the tip of a straight catheter perpendicular to the IA neck. Once packaged into the straight catheter, the coil is directly deployed into the IA. By isolating the IA sac and not modeling the coil advancement step, we believe the simulation does not provide a sufficiently accurate representation of the coil just prior to deployment, i.e. it does not capture the component of strain energy in the coil induced by its bending along the tortuous parent artery.

To capture this strain energy component, in the Improved Technique we explicitly simulate coil advancement in addition to coil packaging and deployment. Instead of modeling advancement of the coil-loaded catheter, we model a curved catheter that is placed directly along the anatomic centerline of the parent artery of the IA. This curved catheter serves as the “path” the coil takes during advancement: after coil packaging, the coil is pushed through the curved catheter to advance it to the IA. Placing the catheter along the anatomic centerline of the parent artery is an approximation. In reality, the catheter will not exactly follow the artery centerline, but instead will minimize its bending energy.

### Virtual coiling workflow

**Fig 2:**
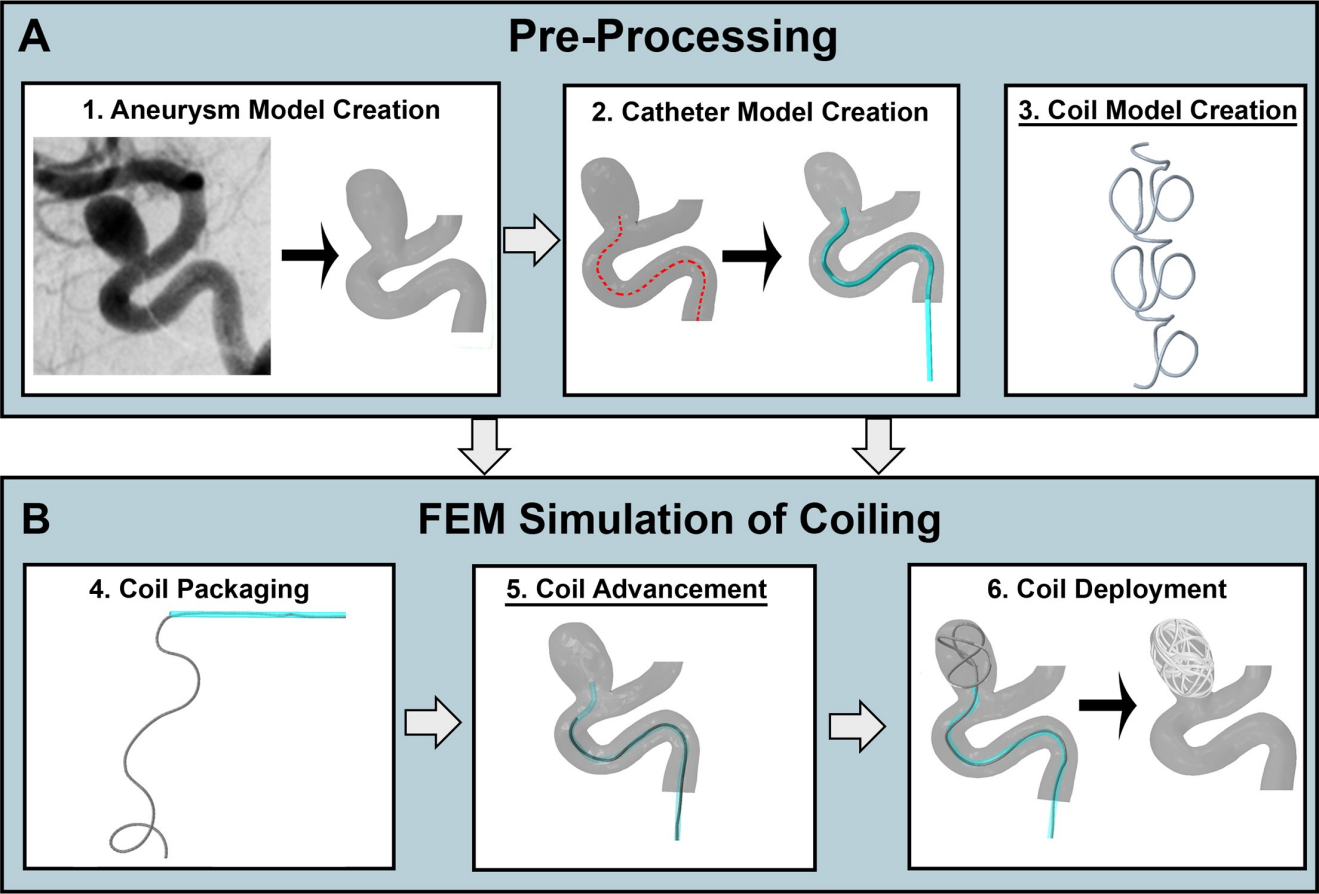
Workflow for virtual coil deployment using FEM. The workflow consists of (A) pre-processing, and (B) FEM simulation. The major improvements (underlined) are in step 3, Coil Model Creation (pre-shape and mechanical properties), and in step 5, Coil Advancement (previously not modeled but now added to capture coil mechanics in tortuous vessels).

The workflow for virtual coiling using FEM is shown in Fig 2 and described below.

#### Pre-processing

The goal of pre-processing is to generate the inputs for the FEM coiling simulations: models of the IA, catheter, and coil(s) (Fig 2A).

1. *Aneurysm Model Creation*–To model coiling in a patient-specific IA, a 3D STL (stereolithographic) model of the IA and its surrounding vasculature is created from either DSA or CTA images of the IA using the Vascular Modeling Toolkit (VMTK - http://www.vmtk.org/) [13], as illustrated in Fig 2A. The clinical images of the IA are segmented using the level set algorithm with isosurface initialization and the marching cubes algorithm in VMTK to create the 3D STL surface.
2. *Catheter Model Creation–*To model a catheter positioned along the parent artery, we first calculate the centerline of the parent artery up to the IA using VMTK and truncate it at the IA orifice, as illustrated in Fig 2A. To better capture the strain energy of the coil immediately before deployment into the IA sac (after coil advancement), we truncate the centerline (and thus parent vessel) upstream of the IA at a length greater than or equal to the length of the longest coil being deployed. Next, to model the catheter, we sweep the centerline with a cylindrical surface in Creo (ver.4., PTC, Boston, MA) and convert the surface to a STL model. The cylindrical radius of the swept catheter is set to be twice the radius of the coil. Finally, the catheter surface is extended perpendicular to and away from the artery inlet upstream of the IA, with a distance equal to the length of the coil.
3. *Coil Model Creation–*The Improved Technique requires a coil model as input, as illustrated in Fig 2A. The strategies for modeling coils were described before.

#### FEM simulation of coiling

We model coil packaging into the catheter, coil advancement to the IA, and coil deployment into the IA, as illustrated in Fig 2B. All simulations are implemented in Abaqus (ver.2016, SIMULIA, Providence, RI). To pull (during packaging) or push (during coil advancement and deployment) the coil in the simulations, we specified displacement boundary conditions on the distal or proximal end of the coil. These boundary conditions were applied using the “smooth step” amplitude specification in Abaqus [14]. This amplitude function ramps up the applied displacement at the beginning of the simulation step and then ramps it down at the end of the step. Specific details of the 3 mechanical steps are described below:

4 *Coil Packaging–*To simulate coil packaging, we first align one end of the coil with the proximal tip of the catheter, and then continuously pull the coil into the catheter by specifying a displacement until it is fully inside. The total amplitude of the displacement is equal to the length of the coil.
5 *Coil Advancement–*After packaging, the coil is pushed along the stationary catheter (along the tortuous parent artery) to advance it to the IA. To do this, a displacement is applied to the proximal end of the packaged coil, with an amplitude equal to the arc-length of the centerline of the catheter.
6 *Coil Deployment–*Once advanced to the IA through the stationary catheter, the coil is pushed out of the catheter to deploy it into the IA sac by applying a displacement until it is fully released from the catheter. To mimic clinical placement of a balloon or stent to prevent coil herniation into the parent artery during coil deployment (especially in wide-necked IAs) [15], an artificial STL surface is modeled across the IA orifice.

Since coiling is mechanically dynamic with many contact interactions (e.g. coil-to-IA contact), we use the explicit dynamics solver in Abaqus for all simulations (Abauqs/Explicit). This Abaqus/Explicit solver uses a lumped mass matrix approximation, along with a central difference rule for time integration [14]. The time step is adjusted automatically throughout the simulation to maintain numerical stability, based upon estimates of the highest frequency of each individual beam element in the model. The estimate of the stable time increment also depends on material damping, which here was taken in the form of Rayleigh damping with both mass (*α*) and stiffness (*β*) proportional contributions. These 2 damping coefficients are determined empirically for each model. To model contact, we use the “general contact” algorithm with the “penalty” method for tangential contact behavior and “hard” contact pressure-overclosure for normal contact behavior. All contact interactions are modeled including coil-to-coil, coil-to-catheter, and coil-to-IA and for each interaction, a specific friction coefficient is specified. During simulations, the catheter and IA are kept rigid. To model deployment of multiple coils into the same IA, the 3 mechanical steps are simulated sequentially for each coil. We assume that the effect of gravity on the coil is negligible compared to the effect of other forces in the model, such as the internal forces in the coil and those due to contact interactions, and thus do not model it in the Improved Technique, nor did we model it in the Original Technique.

### Experimental method to evaluate simulations

**Fig 3:**
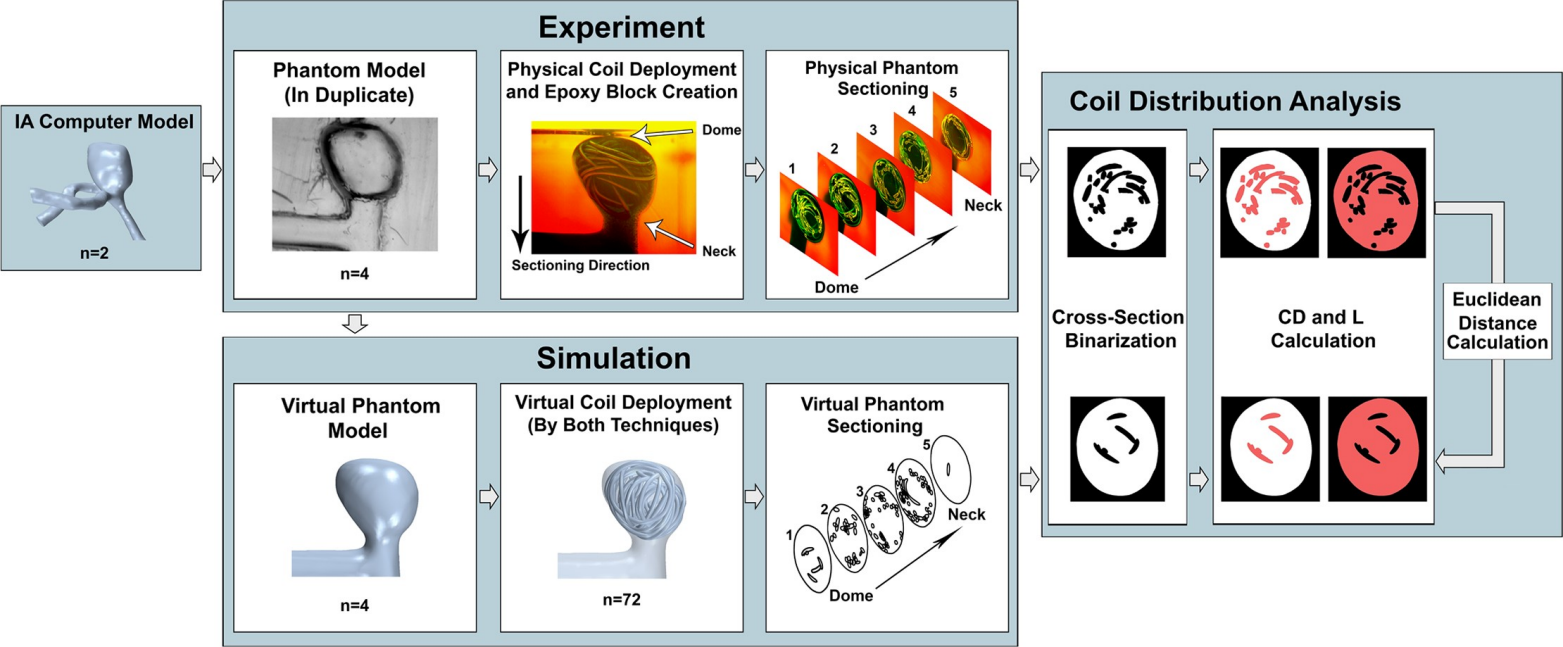
Flowchart of our experimental approach. From Left to Right, computer models of 2 IAs were used in this study as testbeds. Experiment: Based on the IA models, 4 silicone phantoms (duplicates of each IA) were fabricated, coils were deployed into each phantom, and intra-aneurysmal cross-sections were extracted. Simulation: The phantoms were imaged to create virtual models, and coil deployments were virtually recreated using the Original Technique and Improved Technique (9 iterations for each phantom). Cross-sections were extracted matching those in the experiments. Coil Distribution Analysis: Physical and virtual coil distributions were quantified by coil density (*CD*, space occupied by coil–in red in left image) and lacunarity (*L*, gaps between coils–red in right image) from cross-section images. To compare the virtual techniques against experiments, Euclidean distances from the experimental coil distributions to the virtual coil distributions were calculated and evaluated (Coil Distribution Analysis).

To test whether the new modeling strategies result in better accuracy of virtual coil deployments, we physically deployed coils in patient-specific IA phantoms. Then, we conducted simulations to match the experimental conditions using both the Improved and the Original Techniques, extracted coil distributions from both simulations, and compared them against experimental results. A flowchart of the experimental procedure is shown in Fig 3.

#### Patient-specific IA models and phantom fabrication

Two coil-treated patient-specific aneurysms were used to create IA models as testbeds. Under institutional review board approval, we retrospectively obtained the pre-treatment DSA images of the 2 IAs at the Gates Vascular Institute (Buffalo, NY). One aneurysm was a 5 mm paraclinoid internal carotid artery (ICA) IA, the other was a 6 mm anterior communicating artery (ACOM) IA. We segmented their DSA images to create 3D STL models (Fig 3), with surface triangles of 0.2 mm base size. To facilitate phantom fabrication and physical coil deployments, we straightened the inlet parent arteries in both STL models but kept the original aneurysm geometry.

We fabricated 4 optically clear silicone phantoms using a previously developed phantom fabrication method [16]: 2 ICAs (Phantoms I1 and I2), and 2 ACOMs (Phantoms A1 and A2). Since the geometry of an IA phantom may differ from its original STL model, we imaged the phantoms and used those geometries in our simulations, so both virtual and physical coiling used the same IA geometries (Fig 3). The phantoms were imaged using DSA with a Toshiba Infinix C-arm (Canon Medical Systems, Ōtawara, Japan) and the resulting images were segmented to create STL models of the phantoms.

#### Coil deployments in physical phantoms and cross-sectioning

We filled the silicone IA phantoms with water to provide lubrication for coil deployment. A neurosurgeon deployed 1 or 2 bare platinum coils (Axium Prime Detachable Coils, Medtronic, Minneapolis, MN) into the phantoms. The type and size of the coils deployed in each phantom is summarized in Table 1. The primary wire and secondary structure diameters of all the physical coils were *D*_1_ = 0.0381 *mm* and *D*_2_ = 0.2921 *mm*.

**Table 1:** The type and size of coils deployed in each physical and virtual phantom.

	Coil Size D_3_ (mm) x l (cm)	Coil Type
Phantom I1 (ICA)	5 x 10	Axium Prime 3D Bare Platinum Coils
Phantom I2 (ICA)	5 x 10	
Phantom A1 (ACOM)	6 x 20; 5 x 10	
Phantom A2 (ACOM)	6 x 20; 5 x 10	

To preserve deployed coil configuration, the phantoms were drained of water and filled with a low-viscosity epoxy resin (SPI Spurr Formula, West Chester, PA) [17]. Epoxy was injected slowly into each phantom as to not disturb the coils. Once solidified, the phantoms were removed from the silicone and cast into epoxy resin blocks (Fig 3). The epoxied phantoms were oriented in the blocks such that their necks and domes were parallel to one block face and perpendicular to the 4 adjacent faces.

Because we could not obtain 3D coil configuration from clinical imaging, we obtained 2D coil distributions on a sequence of intra-aneurysmal cross-sections by sectioning the epoxy blocks. Each block was sanded in planar cross-sections, 1 mm apart using a belt sander. The block surfaces were polished and imaged after each 1 mm interval. In total, 5 sequential cross-sections were obtained from each phantom, beginning from the IA dome and progressing towards the neck (Fig 3).

#### Modeling the physical coil deployments

We virtually recapitulated the physical coil deployments in the 4 phantoms using both the Original and Improved Techniques (Fig 3). For each phantom, we performed 18 simulations: 9 times using the Original Technique and 9 times using the Improved Technique. We did this to capture the natural variation in the dynamic and random coil deployment process. To represent this variability, we changed the initial conditions of deployment by rotating the coil in 40° increments (up to one full rotation of 360°) coaxially with respect to the catheter before each packaging simulation. This resulted in a total of 72 unique virtual coil deployments (18 per phantom: 9 by the Original Technique and 9 by the Improved Technique).

For all simulations using the Original Technique, we generated mathematical coil pre-shapes having the same dimensions (*D*_3_×*l*) as the 2 different sized coils (Table 1). For the coil’s mechanical properties, we assigned beam elements to have the same elastic moduli as platinum (92%)/tungsten (8%) alloy, *E*_*b*_ = 230 *GPa* and *G*_*b*_ = 82 *GPa* [5, 9]. For all simulations using the Improved Technique, we created coil pre-shapes of the 2 different coils by modeling 2 different sized coil mandrels, using the same coil winding pattern for each coil. Note that because the mandrel design and winding pattern for Axium coils are proprietary, we modeled a generalized mandrel design and winding pattern from a patent for coils by a different coil manufacturer because that patent explicitly describes and illustrates mandrel designs and winding patterns [8]. In addition to “virtually manufacturing” the pre-shapes, we calculated the appropriate elastic moduli of the coil’s beam elements to be *E*_*b*_ = 1.52×10^−3^ *GPa* and *G*_*b*_ = 1.85×10^−3^ *GPa* from the aforementioned spring rigidity Eqs (1–6).

To demonstrate the capabilities of our Improved Technique at simulating coiling in anatomical vascular geometries, we also performed an additional coiling simulation in the original anatomical STL model (not the phantom model) of the ICA aneurysm, which contained its tortuous parent artery. Using the Improved Technique, we simulated the complete deployment procedure of a 5 mm x 20 cm coil into this IA. Note that we used a longer coil (20 mm length) for this simulation in the anatomical ICA than the one used in the experiments (10 mm length) in order to demonstrate coil advancement through a long and tortuous parent artery. For comparison, we also performed the same coil deployment using the Original Technique (also in the anatomical STL model, but with the anatomical IA sac isolated from the parent artery).

All FEM simulations were performed on a desktop computer with 2 Intel Xeon processors (CPU E5-2687W 0 @ 3.10GHz) and 128 GB RAM. Simulations were run in serial with no parallel processing, and average simulation times in the 4 phantoms for each technique were reported in hours. In simulations for both techniques, we specified coil-catheter contact interactions as frictionless. Coil-to-coil contact was modeled with a 0.2 coefficient of friction to model lubricated platinum-to-platinum contact [4]. We specified a 0.6 coefficient of friction for coil-to-IA contact to model the platinum-to-silicone contact, based on experimental measurements of the coefficient of friction of silicone vascular phantoms by Ohta et al. [18]. In this study, we found *α* = 1 *s*^−1^ and *β* = 10^−6^ *s* to be appropriate for damping in all simulated coil deployments.

#### Virtual phantom cross-sectioning

From the simulation results, we extracted 2D cross-sections in the virtual coiled phantoms to match those in the experiments. This was done by aligning the virtual phantoms with cross-section images of the physical phantoms in Star-CCM+ (Siemens, Melville, NY), using the outlines of the parent artery and IA sac as reference.

### Comparisons of results from the 2 FEM techniques

Coiling simulations by both the Original and Improved Techniques as well as their resulting deployed coil configurations were compared against experiments, both qualitatively and quantitatively.

#### Quantification of coil distributions–coil density and lacunarity

Being deployed into the IA sac, coils create a three-dimensional porous medium with varying pore sizes and shapes [19]. Because 3D coil configuration (and thus the pore spaces around the coils) cannot be obtained from imaging, we used 2 surrogate metrics defined in 2D to characterize and quantify coil distribution on the phantom cross-sections. For each phantom, the physical and virtual cross-section images were binarized and the outline of the IA sac in the first cross-section was used as a mask (Fig 3). We characterized and quantified coil distribution on the binarized phantom cross-sections using 2 metrics: coil density (*CD*) and lacunarity (*L*) (Fig 3).

Coil density quantifies how much of a cross-section is occupied by coil. We calculated *CD* by dividing the pixel area of coils (black) in a binarized cross-section by the total pixel area of the interior of the mask in that cross-section (white + black) [20]. Lacunarity quantifies how coils are spatially distributed by measuring distributions of gaps (or spaces) between coils in the cross-sections (white space) [21]. Coil distributions with low *L* are spatially homogeneous with similar gap sizes whereas coil distributions with high *L* are spatially heterogeneous with varying gap sizes. We calculated *L* using FracLac (https://imagej.nih.gov/ij/plugins/fraclac/fraclac.html) in ImageJ (https://imagej.nih.gov/ij/). The “sliding box” algorithm in FracLac was used to calculate *L* [20].

#### Quantitative comparison between experimental and simulated coil distributions

For comparison purposes, we characterized each deployed coil configuration on the 2D cross-sections using a combination of *CD* and *L*. To compare techniques in the 4 phantoms, coil distributions on the 5 cross-sections from the Improved Technique, Original Technique, and the experiments were plotted together in a 2D coordinate system (i.e. (*CD*,*L*)). For convenience of comparing the 2 techniques visually, we also plotted the mean point of the 9 deployments by the Improved Technique and the mean point of the 9 deployments by the Original Technique.

To obtain an aneurysm-averaged coil configuration in each phantom, we averaged the values of *CD* and *L* of each deployment (physical or virtual) over the 5 cross-sections. To place *CD* and *L* on the same scale, we standardized all of the aneurysm-averaged points in each phantom such that the mean and standard deviation for both *CD* and *L* of all the points were 0 and 1, respectively. For each of the 4 phantoms, standardized aneurysm-averaged *CD* and *L* were plotted separately and analyzed.

To quantitatively assess our coiling techniques, we calculated Euclidean (“straight-line”) distances between standardized aneurysm-averaged experiment points and each virtual point on the *CD*-*L* plane. Euclidean distance (*d*) was calculated by:

$$d=\sqrt{{\left({CD}_{simulation}-{CD}_{experiment}\right)}^{2}+{\left(L_{simulation}-L_{experiment}\right)}^{2}}$$ (7)

Three specific distances were calculated using Eq (7) and evaluated: *d*_*Min*_, the smallest of the distances between the experiment point and any of the 9 virtual points (for each technique), *d*_*Max*_, the largest of the distances between the experiment point and any of the 9 virtual points (for each technique), and *d*_*Avg*_, the distance between the experiment point and the mean of the 9 virtual points (of each technique).

To compare simulation results across the 4 phantoms, we grouped the 4 *d*_*Min*_, the 4 *d*_*Max*_, and the 4 *d*_*Avg*_ of both techniques, and performed univariate tests in SPSS (ver.25, IBM, Armonk, NY) to determine if *d* was significantly different between the techniques. To do so, we first tested the groups of distances for normality by performing Shapiro-Wilk tests. Because we found all groups to be normally distributed, we tested their equality of variances using Levene’s tests, and performed Student’s t-tests with equal variances assumed where appropriate. Euclidean distance (*d*) was reported as mean ± standard deviation and *d* was determined to be significantly different between techniques if p < 0.05.

## Results

### Simulation of complete coil deployment procedure in an anatomical vascular geometry

**Fig 4:**
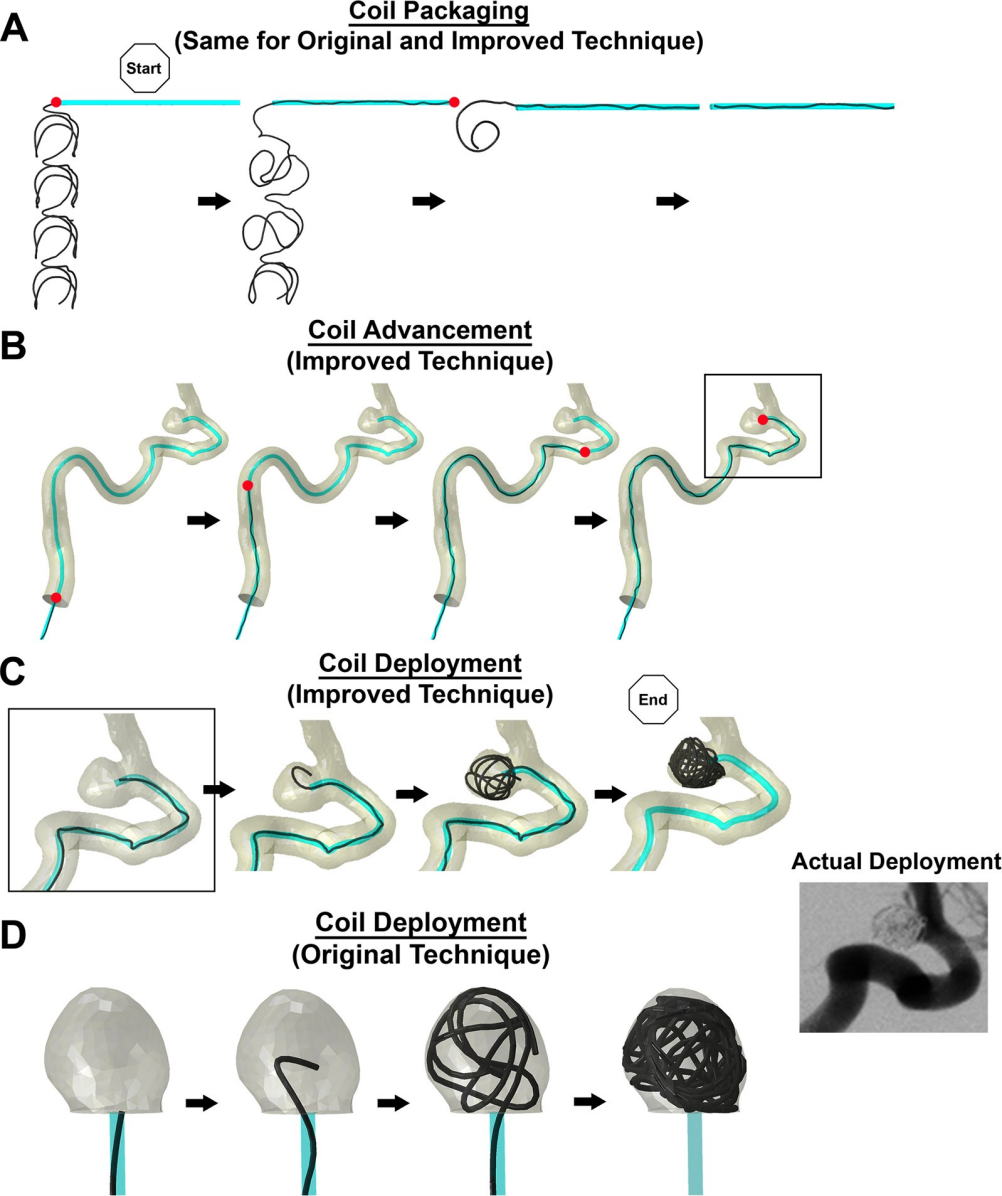
The 3 mechanical steps of virtual coiling. (A) Coil Packaging: The coil, in its pre-shape configuration, was pulled continuously into the proximal end of the catheter until it was straightened (red dot = distal tip of the coil). (B) Coil Advancement: The coil was continuously pushed in the catheter (positioned at the parent artery centerline) until it reached the IA. (C) Coil Deployment (Improved Technique): The beginning of coil deployment happens directly after advancement (same geometry in the “box”). For both C and D, the coil was continuously pushed along the catheter into the IA sac until it was completely deployed, ending the FEM simulation. The stable time increment was approximately Δ*t* = 3 *μs* throughout the simulation of the 3 mechanical steps. (D) Coil Deployment (Original Technique): Coil deployment by the Original Technique occurs without advancement through the catheter, only occurring at the neck of the IA. As shown in the DSA image, the Improved Technique better resembled coil deployment in the actual IA than the Original Technique.

To test the capability of our Improved Technique at simulating the complete coil deployment procedure in an anatomical vascular geometry, we simulated coiling in the original STL model of the patient-specific ICA aneurysm. Snapshots of the simulation, shown in Fig 4, demonstrate successful implementation of the advancement phase of the simulation whereby the coil was able to navigate the tortuous parent artery. Compared to coil deployment by the Original Technique, coil deployment by the Improved Technique better resembled the actual coil deployment in the ICA.

### Qualitative comparison against experiment

**Fig 5:**
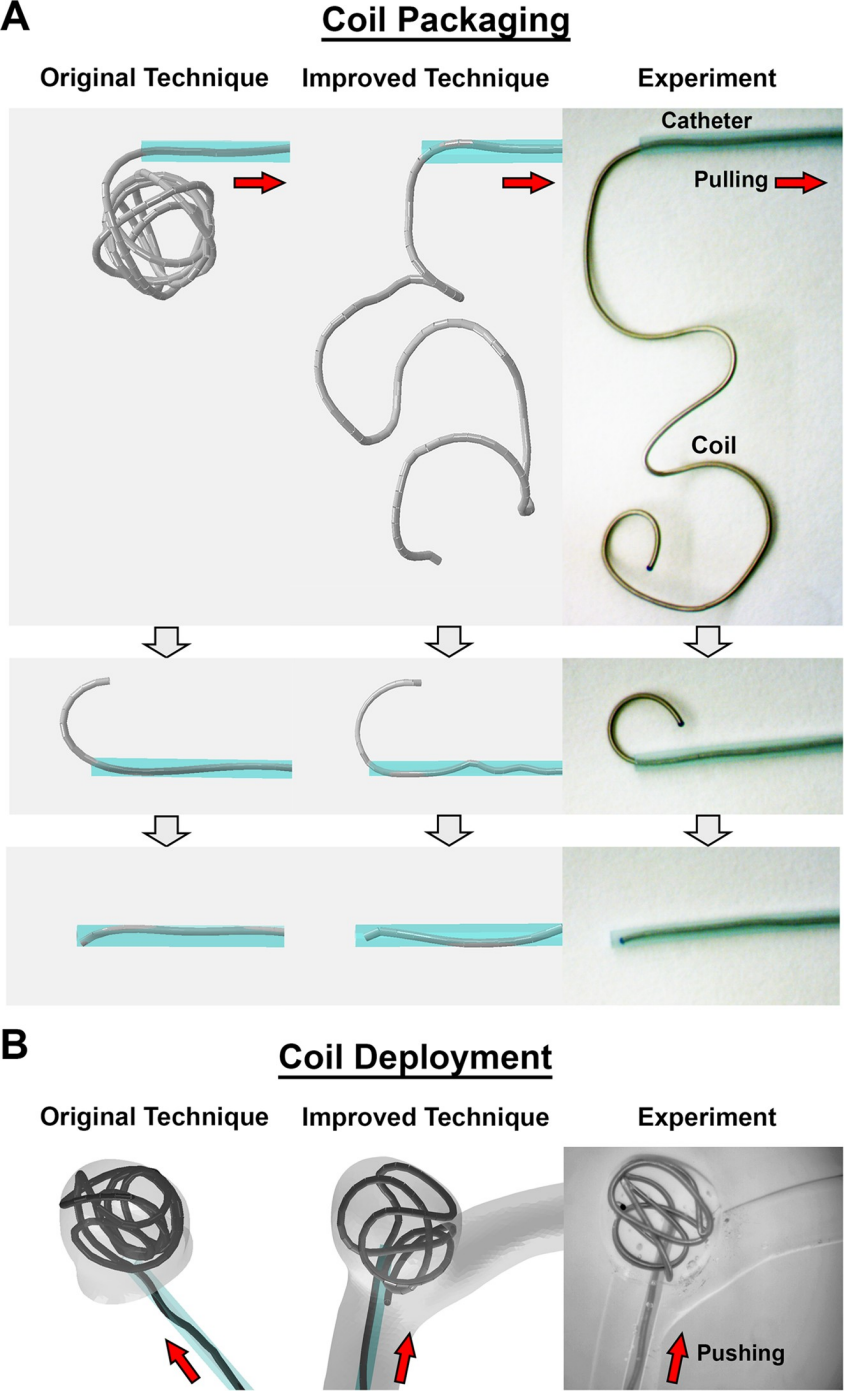
Coil packaging and deployment in experiment and simulation. (A) Coil packaging is the dynamic process in which the coil is pulled into the catheter from its pre-shape. Qualitatively, the packaging simulation by the Improved Technique better resembles the experiment than the packaging by the Original Technique. However, it should be noted that gravity was not considered in simulations. (B) An example of coil deployment is shown in physical and virtual Phantoms I2. Catheter placement and coil advancement by the Improved Technique resembles the experiment more than the Original Technique.

Sequential images of coil packaging and deployment in experiment and by both simulation techniques are shown in Fig 5, using Phantom I2 as example. Coil packaging by the Improved Technique better resembled the experimental coil packaging than the Original Technique (Fig 5A). In addition, the position of the catheter in the Improved Technique better resembled the position of the catheter in experiment than in the Original Technique (Fig 5B).

**Fig 6:**
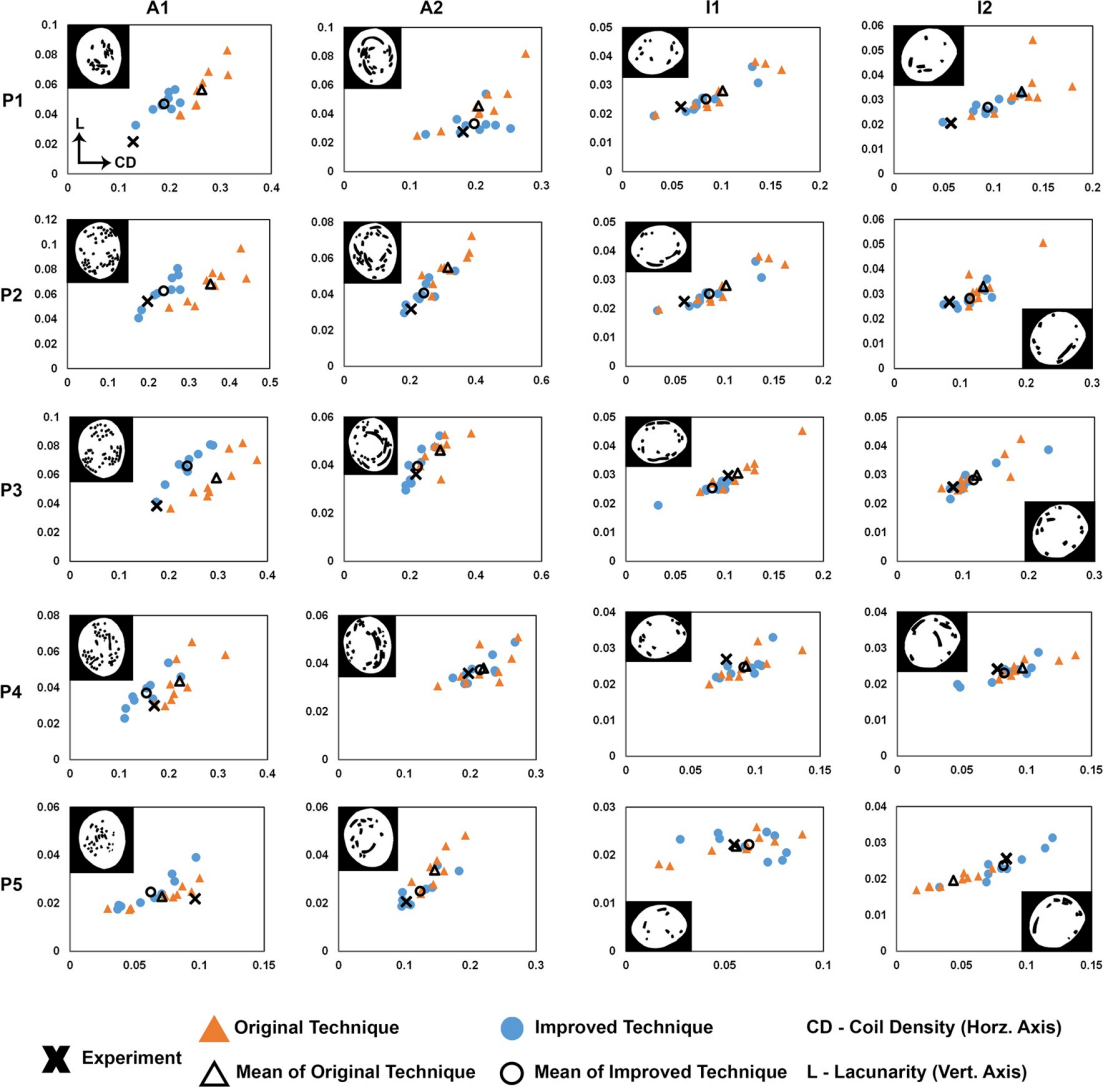
Quantification of coil experimental and computational results on cross-sections in aneurysm Phantoms A1, A2, I1, and I2. The binarized image of each experimental cross-section is shown in its corresponding graph. Graphs labeled P1-P5 show the raw values for lacunarity (*L*—vertical axis) vs. coil density (*CD*—horizontal axis) measured on each phantom cross-section (1–5), whereby experimental points are shown by a cross, and individual virtual coil deployments are represented by circles (Original) or triangles (Improved). The mean of the virtual coiling results (9 realizations by each technique) are represented by hollow circles (Original) or hollow triangles (Improved). Note that the upper bounds of the vertical and horizontal axes of each graph differ in order to fit the data points tidily in each graph. We observed that in all cross-sections, the range of both virtual coiling techniques lies near the experiment point. Furthermore, in the majority of cross-sections, the mean of the Improved Technique was closer to the experiment than the mean of the Original Technique.

Fig 6 shows binarized images of cross-section planes (P1-P5) from the 4 physically coiled IA phantoms, together with the *CD*-*L* graphs for these cross-sections. The range of points of both virtual coiling techniques were near the experiment points for the same phantom and the same cross-sections (P1-P5) of all 4 phantoms. The Improved Technique generated coil distributions (in terms of cross-sectional *CD* and *L*) that were closer to the experiments. This was discerned by visually examining the distances between the mean of either coiling technique and the experimental data points: in the majority of cross-sections, the distances were smaller for the Improved Technique.

### Quantitative comparison against experiment: coil density and lacunarity

**Fig 7:**
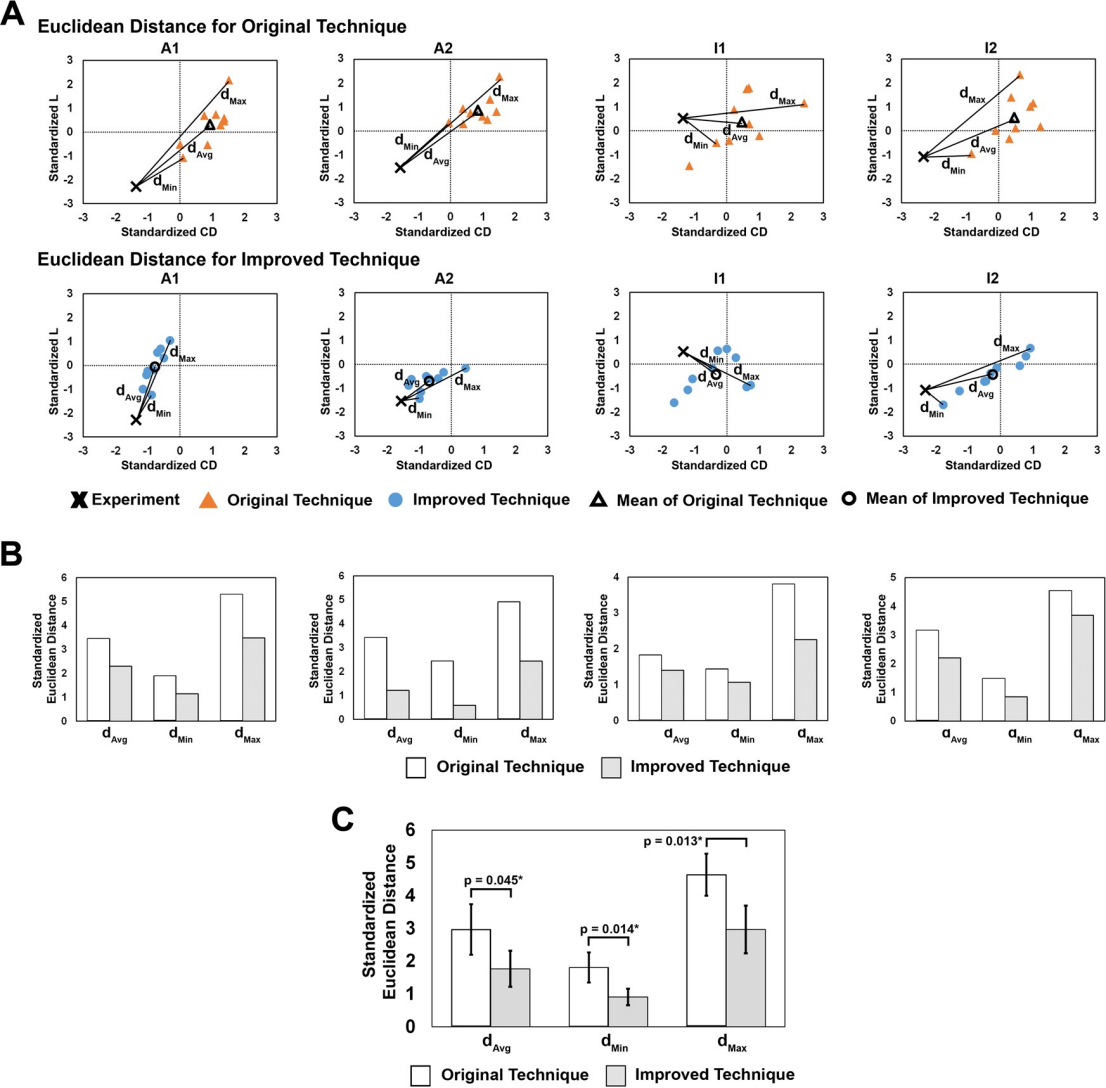
Euclidian distances from experimental to computational results of both techniques in the standardized, aneurysm-averaged *CD*-*L* plane. (A) Example of the 3 Euclidean distances evaluated for comparison of both FEM techniques in the 4 phantoms, namely *d*_*Avg*_, *D*_*Min*_, and *d*_*Max*_. Graph axes represent “standard deviations”, where the origin (0,0) is the mean of standardized *CD* and *L*. (B) Bar graphs of the 3 standardized Euclidean distances calculated for both techniques in the 4 phantoms. In all 4 phantoms, *d*_*Avg*_, *d*_*Min*_, and *d*_*Max*_ were smaller for the Improved Technique than for the Original Technique. (C) Results of the univariate tests to compare Euclidean distances between techniques across the 4 phantoms. The average *d*_*Avg*_, *d*_*Min*_, and *d*_*Max*_ in the Improved Technique had significantly smaller Euclidean distance than the average distance in the Original Technique (significance indicated by asterisk and the p-values for each test are reported).

Fig 7 shows the quantitative results of the 3 evaluated Euclidean distances (i.e. *d*_*Min*_, *d*_*Max*_, and *d*_*Avg*_) measured from experiment to the computational results of both techniques. Fig 7A shows standardized aneurysm-averaged *CD* and *L* for both techniques for each of the 4 phantoms. The graphs show that the Improved Technique produced coil deployments, in terms of standardized *CD* and *L*, closer to the experimental realizations in the 4 phantoms than the Original Technique.

Fig 7B shows bar graphs of the 3 evaluated Euclidean distances for the 2 virtual coiling techniques in the 4 phantoms. It is clear that all 3 are consistently smaller in the Improved Technique than in the Original Technique in all 4 phantoms. In other words, regardless of what distance is considered, the Improved Technique was closer to the physical coil deployments than the Original Technique.

A bar graph of *d*_*Min*_, *d*_*Max*_, *d*_*Avg*_ averaged across the 4 phantoms for both simulation techniques is shown in Fig 7C. Average Euclidean distance was significantly smaller for the Improved Technique than for the Original Technique (*d*_*Min*_: *p* = 0.014, *d*_*Max*_: *p* = 0.013, *d*_*Avg*_: *p* = 0.045). This result indicates that the Improved Technique simulated coil deployments that were significantly closer to the physical deployments than deployments by the Original Technique in terms of *CD* and *L*.

**Fig 8:**
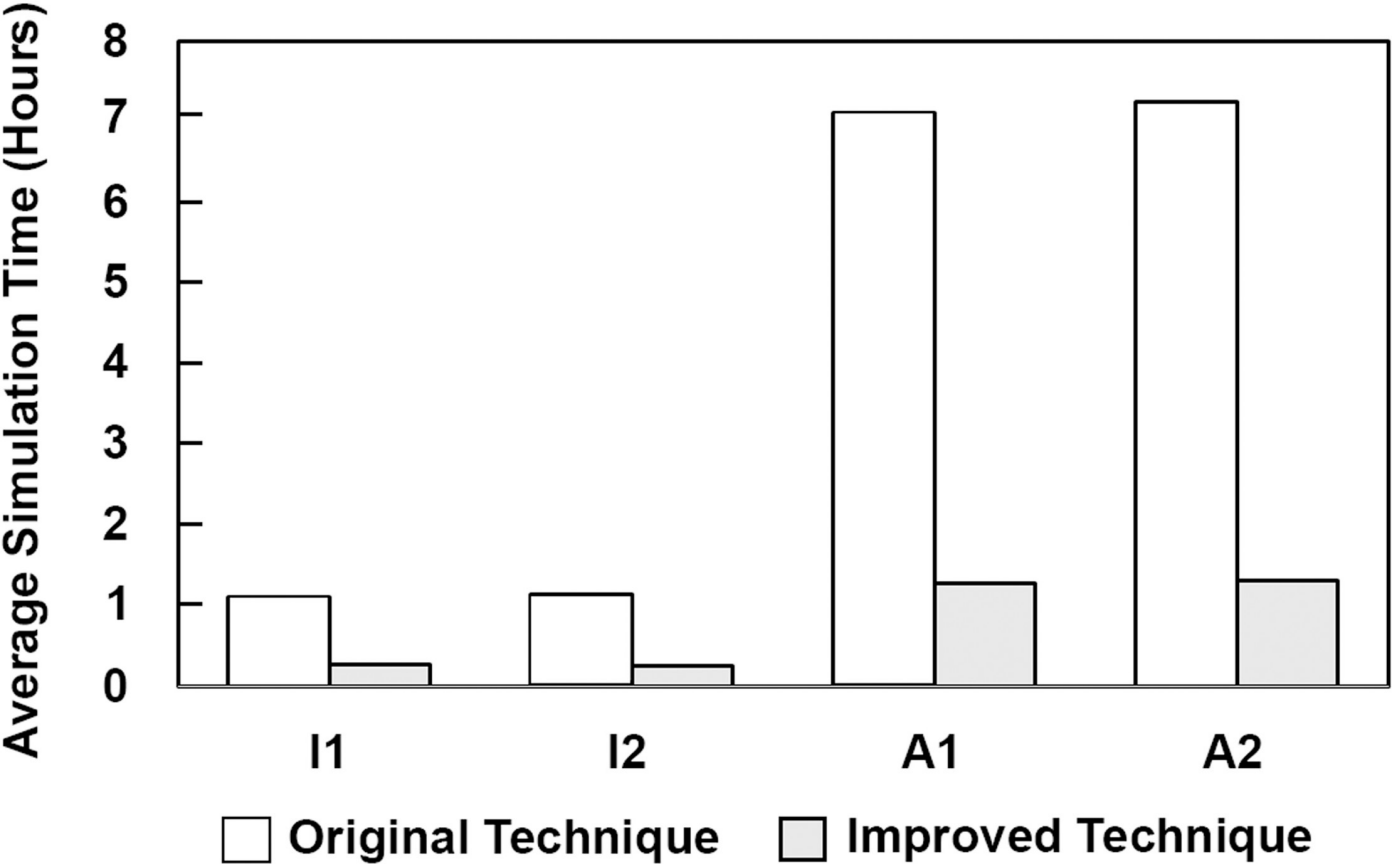
Average simulation times of the 9 virtual deployments by each FEM technique. In all 4 phantoms, the average simulation time of the Improved Technique was smaller than for the average time of the Original Technique.

In addition to simulating coil configurations closer to the physical deployments than the Original Technique, the Improved Technique was also found to be more computationally efficient than the Original Technique. Fig 8 shows a graph of the average simulation time of the 9 virtual deployments by each technique in the 4 phantoms. In all 4 phantoms, the average simulation time was shorter for the Improved Technique than for the Original, with the largest difference in simulation times being in Phantoms A1 and A2.

## Discussion

We implemented 3 advanced features in our FEM coiling technique: coil pre-shape created from mandrels, coil mechanical stiffness properties based on springs, and coil advancement through the parent artery. Experimental results show that these new modeling strategies produce coil deployments that are more realistic. Quantitatively, our data demonstrate that the Improved Technique is more accurate and efficient than the Original Technique. This study highlights the importance of including coil-specific physical properties and mechanical coiling steps to accurately model coiling.

### The 3 new strategies

The first new strategy implemented in this study was the virtual manufacturing of the coil pre-shape by explicitly modeling a mandrel and simulating mechanical winding of the coil around it to obtain its pre-shape. To our knowledge, this is first time such a strategy has been employed. Previous coiling techniques (including our own) followed the approach of Babiker et al. and used generalized mathematical curves to model coil pre-shapes [3–5, 22], which, as shown in this study, do not result in accurate deployed coil configurations. Furthermore, the previous strategies cannot model coil pre-shapes from different manufacturers, whereas the Improved Technique could be tailored for different types of coils for *a priori* treatment evaluation by creating manufacturer-specific mandrels and winding patterns. However, because pre-shapes of commercialized coils are typically proprietary, we admit that a more practical application of our novel modeling strategy could be in creating and evaluating new coil pre-shape designs.

The second new strategy, based on the approach by Otani et al. [5], was the adoption of spring-like properties to better reflect the secondary structure of coils in the beam elements. Based on our data, this change (in conjunction with changing the beam element formulation) made the mechanical rigidity of the coil’s beam elements (e.g. axial and flexural) approximately 5 orders of magnitude smaller than those in the Original Technique, which models coils using “stiff” mechanical properties based on platinum wires. Like mandrel winding, this improvement can also be tailored for different types of coils with different “softness” [9], since it uses physical characteristics of a coil’s helical spring-like structure and material composition to determine its mechanical properties. While this improvement made the modeled coils much “softer” in the Improved Technique than in the Original Technique and presumably closer to the mechanical properties of actual coils, we admit that additional experimental and computational analyses are required to test this.

In addition to improvements in coil physical properties, our third new strategy was the implementation of coil advancement to the IA. Previous techniques either neglected this step by directly deploying the coil into an isolated IA sac [3–6], or simplified this step by removing a majority of the parent artery [22]. We do not believe an isolated IA sac or drastically shortened parent artery provides a sufficiently accurate representation of the state of deformation of the coil just prior to deployment into the IA. Consequently, in our Improved Technique, we included coil advancement to more accurately capture the stored strain energy that the coil would possess by bending in the parent artery. This improvement can be highly significant in patient-specific IA cases with tortuous vessels. This provides utility for future applications of the FEM technique because it affords the ability to simulate coiling in a wide array of tortuous patient-specific vascular geometries.

### Experimental evaluation

Validating coiling simulations is challenging, primarily because current clinical imaging cannot resolve the configuration of deployed coils, making true *in vivo* comparisons impossible. In this study, we developed a novel *in vitro* approach that offers several advantages over previous published validation strategies. Specifically, prior efforts (including our own [3]) simulated coil deployments in idealized or patient-specific IA models, and indirectly compared the simulation results to histology of coiled rabbit aneurysms [3, 4, 23, 24]. However, such comparisons are inherently limited, since only qualitative similarities between simulated coil configurations and histological data can be drawn, and therefore only simple relationships can be gathered, for example, the association between increasing packing density and more homogeneous coil distribution [23].

In our approach, several strategies were incorporated to overcome challenges of previous validation efforts. First, to enable direct comparison of simulation and experimental results, coils were deployed in patient-specific IA phantoms *in vitro*, and then numerically recapitulated using our virtual coiling techniques. Second, each treatment scenario was simulated multiple times by changing the initial conditions in the simulations to account for the intrinsic variability of actual coil deployments. Third, to characterize deployed coil configuration for validation, sequential 2D cross-sections were extracted from phantoms and coil distributions were quantified on these planes using 2 geometrical metrics. This strategy is feasible for both experimental and simulated deployments and is analogous to obtaining coil distribution on 2D histological sections. And lastly, we calculated Euclidean distances between the experimental and virtual deployments in the standardized *CD*-*L* plane to quantitatively evaluate our coiling techniques. Overall, these improvements enabled the simultaneous quantitative evaluation of both our FEM coiling techniques, and gave us increased confidence in the reliability of our validation results.

### Modeling improvements translated into more accurate and efficient simulations

Based on experimental results, we showed that the 3 modeling improvements translated into increased accuracy of our FEM coiling simulations. The Improved Technique generated more accurate coil distributions that were closer to the experimental data than the Original Technique (Fig 7), as judged by Euclidean distances in the *CD*-*L* plane. These smaller distances mean that the Improved Technique generated deployed coil configurations that were more geometrically similar to experiments (in terms of the coil’s spatial density (*CD*) and distribution (*L*)) than those generated by the Original Technique. This suggests that the 3 new strategies do make the virtual coil’s physical properties and mechanics more realistic.

In addition to better accuracy, the Improved Technique was also shown to be more computationally efficient. Our data show that it was approximately 5 times faster at simulating coiling in the 4 virtual phantoms than the Original Technique (Fig 8). We suspect this was due primarily to the lower stiffness of the coil’s beam elements in the Improved Technique. Decreasing stiffness increases the stable time increment in explicit dynamic simulations, thereby shortening overall simulation run times [25].

### Implications for the field and future directions

The results of this study have implications for future development of virtual coiling techniques and their application in the clinic to predict the effects of coil treatments and long-term outcomes of patient-specific IAs *a priori*. Since coils induce thrombotic occlusion of an IA by modifying intra-aneurysmal flow, treatment outcomes may depend on both the deployed coil configuration and the post-treatment aneurysmal hemodynamics. We submit that more realistic and accurate coiling simulations should generate more accurate hemodynamic results, since inaccuracy in representing geometry in CFD (e.g. the IA and deployed coil configuration) could lead to unreliable flow simulations [26]. Because we found that the Improved Technique simulated physical coil deployments more accurately than the Original Technique, we suspect that it would also generate more accurate hemodynamic results. However, future experimental studies are required to show this.

In addition to being accurate, a virtual coiling technique must also be efficient to be able to fit into fast-paced clinical workflows for *a priori* treatment evaluation. While we showed in this study that the Improved FEM Technique is more efficient than our Original Technique, it still took an average of 1 hour to simulate deployment of just 2 coils on a desktop computer (Fig 8). Thus, our FEM technique (and FEM techniques in general) are currently too inefficient for clinical implementation. Several other virtual coiling techniques have been developed that are more efficient than FEM, but potentially less accurate. At the simplest level, the porous media technique achieves efficiency by representing coils implicitly as a porous region in the IA sac [27]. While efficient, this technique prohibits localized flow analysis around the coils, and a previous study showed that it simulates inaccurate post-treatment hemodynamics [28]. A step above porous media in terms of complexity are techniques that explicitly model some coil geometric properties (e.g. *l* and *D*_2_) and can simulate coil deployment in a matter of minutes [29–31]. For example, one technique developed by Morales et al., called dynamic path planning, models coil deployment efficiently by using mathematical constraints instead of mechanics [30]. While these techniques are potentially efficient enough for clinical workflows, their accuracy at simulating realistic deployed coil configurations is questionable because they do not account for the tertiary pre-shape or mechanics of coils. We showed in the current study that it is important to incorporate the physical properties of coils and simulate their mechanics to achieve more accurate coil deployments, so future “hybrid” techniques should aim to incorporate these features while maintaining efficiency.

### Limitations

Our study has several limitations. First, we did not consider the interaction of blood with coils in our FEM-based coiling technique, nor did we replicate pulsatile flow conditions in our *in vitro* coil deployments. However, *in vivo*, adverse events like coil compaction can occur when coils cannot withstand the pulsatile forces of blood flow [32]. Thus, future studies should include the interaction of coils with flow during their deployment to obtain more realistic coil configurations. Second, we only considered coil deployments in 2 patient-specific IA geometries, and characterized the deployments using only 2 metrics on a limited number of planar cross-sections. Therefore, coil deployments in greater numbers of IA geometries with more advanced techniques to extract and characterize deployments should be considered. Lastly, it is not possible to discern the contribution of each of the 3 new modeling strategies to increasing the accuracy of the FEM technique, since all 3 were employed simultaneously. Future experimental investigations are needed to determine which properties/mechanics have the most impact on the accuracy of virtual coiling simulations.

## Conclusions

In this study, we enhanced the modeling strategies in our FEM coiling technique and experimentally tested whether they resulted in more accurate coil deployments. The improved FEM technique was more accurate and efficient than the original FEM technique. The results of this study highlight the importance of incorporating coil-specific physical properties and mechanics in FEM simulations of endovascular coiling of intracranial aneurysm for accuracy.
